# Colloidal silica-induced hypersensitivity: myth or reality topics: clinical cases/case series: non-immediate elayed T cells IgG

**DOI:** 10.1186/2045-7022-4-S3-P79

**Published:** 2014-07-18

**Authors:** Nadia Ben Fredj, Najeh Ben Fadhel, Zohra Chadly, Naceur Boughattas, Karim Aouam, Amel Chaabane

**Affiliations:** 1 Faculty of Medecine, Pharmacology department, Tunisia

## Background

Colloidal silica is an inorganic substance widely used as excipient in pharmaceutical preparation. It acts as a vehicule to increase the absorption of many oral drug formulations. Many excipients have been reported to induce drug hypersensitivity such as tartrazine, aspartame, benzalkonium chloride, sodium metabisulphite, propyl gallate. However, colloidal silica has never been reported to induce hypersensitivity. We report herein a case of colloidal silica-induced hypersensitivity confirmed by a positive patch test.

## Case report

A 40-year-old patient was prescribed diclofenac (Voltarene® 50 mg enteric coated tablet, Novartis) for a right goneitis. On the second day of treatment, he presented a generalized maculo-papular eruption with neither fever nor lymph node swelling. Laboratory findings showed eosinophilia (500 cells/µL) with normal hepatic and renal function. Voltarene® was discontinued and the symptoms had resolved one week later. One month after total recovery, a patch test to diclofenac (Voltarene®, 50 mg enteric coated tablet, Novartis) was performed on the back of the patient, and was positive. In order to investigate cross reactivity, we performed a patch test to other non steroidal anti-inflammatory drugs (NSAID), i.e; piroxicam (Piroxen®), ketoprofen (Oki®), indometacine (Indocid®), tiaprofenic acid (Surgam®), acetyl salicylic acid (Aspegic®). These tests were positives only for piroxicam, ketoprofen and indometacine. When examining different NSAID formulations used in patch testing, we found that colloidal silica was present in tablets of voltanene®, Piroxen®, Oki®, Indocid® and was absent in Surgam® and Aspegic®. So that, a patch test to colloidal silica was performed (10% in petrolatum) and was positive at 48h reading. A control patch test was conducted in three healthy controls following to the same procedure and was negative in all of them.

## Conclusion

To the best of our knowledge, this case is the first to describe a skin hypersensitivity induced by colloidal silica confirmed by a positive patch test.

